# Osteoradionecrosis After Mandibulotomy and Marginal Mandibulectomy in Patients With Oral Cancer

**DOI:** 10.7759/cureus.33628

**Published:** 2023-01-10

**Authors:** Sombat Wongmanee, Adit Chotipanich

**Affiliations:** 1 Department of Otolaryngology, Head and Neck Surgery, Chonburi Cancer Hospital, Chonburi, THA

**Keywords:** hiv aids, buccal cancer, floor of mouth cancer, tongue cancer, mandibulectomy, osteoradionecrosis, oral cancer, mandibulotomy

## Abstract

Objectives

Osteoradionecrosis is one of the most severe complications in patients with head and neck cancer, which is characterized by persistent exposed and devitalized bone without proper healing after radiation. The extent to which mandibulotomy and marginal mandibulectomy influence the occurrence of osteoradionecrosis remains unclear. This study evaluated the incidence and risk factors for developing osteoradionecrosis of the mandible after oral cancer treatments.

Methods

A retrospective study was performed to analyze medical records of patients who underwent surgery and postoperative radiotherapy for oral cancers from 2009 to 2019 at a tertiary care hospital. Patient characteristics, incidence, and risk factors for developing osteoradionecrosis were reviewed. Comparisons between continuous and categorical data were performed using t-test and Chi-squared test. Cox regression analysis was used to assess the association between factors and the development of osteoradionecrosis.

Results

Among the 61 patients included in the study, osteoradionecrosis of the mandible occurred in 9 of 32 (28.1%) patients who underwent mandibular surgery during oral cancer resection (marginal mandibulectomy and/or mandibulotomy) and 2 of 29 (6.9%) patients without mandibular surgery.

The development of osteoradionecrosis was significantly associated with performing mandibular surgery (hazard ratio 4.64, 95% confidence interval: 1.002, 21.5) and HIV infection (hazard ratio 8.53, 95% confidence interval: 2.2, 33.3). In the subgroup analysis of mandibular surgery, the development of osteoradionecrosis significantly increased in patients undergoing mandibulotomy (hazard ratio 6.62, 95% confidence interval: 1.3, 34.8) but not in patients undergoing marginal mandibulectomy (hazard ratio 3.56, 95% confidence interval: 0.6, 22.0).

The analysis also showed that concurrent chemoradiation, radiation doses ≥ 60 Gy, and smoking were potential risk factors for the development of osteoradionecrosis, but none of these factors were statistically significant.

Conclusion

Our findings suggest that mandibular surgery is a significant risk factor for the development of osteoradionecrosis in patients with oral cancer. Further studies including larger population sizes are required to verify these findings.

## Introduction

Oral cancer is a common type of head and neck cancer, which is reported to be the fifth most common cancer among countries in Southeast Asia and ranks among the top ten dominant cancers in Thailand [1].

While surgical treatment and postoperative radiotherapy are the mainstay treatments for oral cancer, surgical management of these cancers is often more challenging because of the small area of the mouth and adjacent bony structures, which limit surgical exposure. Adequate surgical exposure and en-bloc resection are crucial to ensure oncological safety [2].

Mandibulotomy is an approach that divides the mandible to gain surgical access to the oral cavity and oropharynx. Although this technique requires repair of the mandible afterwards, it provides excellent exposure to the submental area and posterior part of the mouth [3]. Complications of mandibulotomy in the radiation field include malunion, nonunion, malocclusion, dental complications, and potential osteoradionecrosis [4].

Mandibulectomy is a procedure used to eradicate a disease that involves the mandible. Marginal mandibulectomy involves resecting a portion of the lingual cortex or the alveolar ridge of the mandible as part of the surgical margin. This technique is indicated for oral cancers that abut or minimally erode the mandible without gross invasion. A decrease in mandibular bone thickness after resection may increase the risk of developing fracture and osteoradionecrosis [5].

Several risk factors, such as poor dental health, post-radiation dental extractions, and radiation doses, have been reported to be associated with the development of osteoradionecrosis [6]. However, the relationship between mandibular surgery and osteoradionecrosis after oral cancer treatment remains unclear due to a lack of studies [4,7,8].

The aim of this retrospective study was to provide objective data on the role of mandibular surgery as a risk factor for the development of osteoradionecrosis in patients with oral cancer. The characteristics, incidence, and risk factors for developing osteoradionecrosis after oral cancer treatments were analyzed.

## Materials and methods

This retrospective study reviewed surgical cases performed in the Department of Otolaryngology at the Chonburi Cancer Hospital, Thailand. Data regarding surgical techniques, clinical course, and complications were obtained from patients’ medical records.

The inclusion criteria were: new diagnosis of oral cancers with at least three-year survival and receiving postoperative radiotherapy. The exclusion criteria were: receiving radiotherapy prior to surgery; premature discontinuation of treatment; performing segmental mandibulectomy with or without free flap reconstruction; or requiring revision surgery due to major surgical complications. Patients with a daily frequency of smoking were classified as smokers, regardless of duration.

Surgical technique

**Figure 1:**
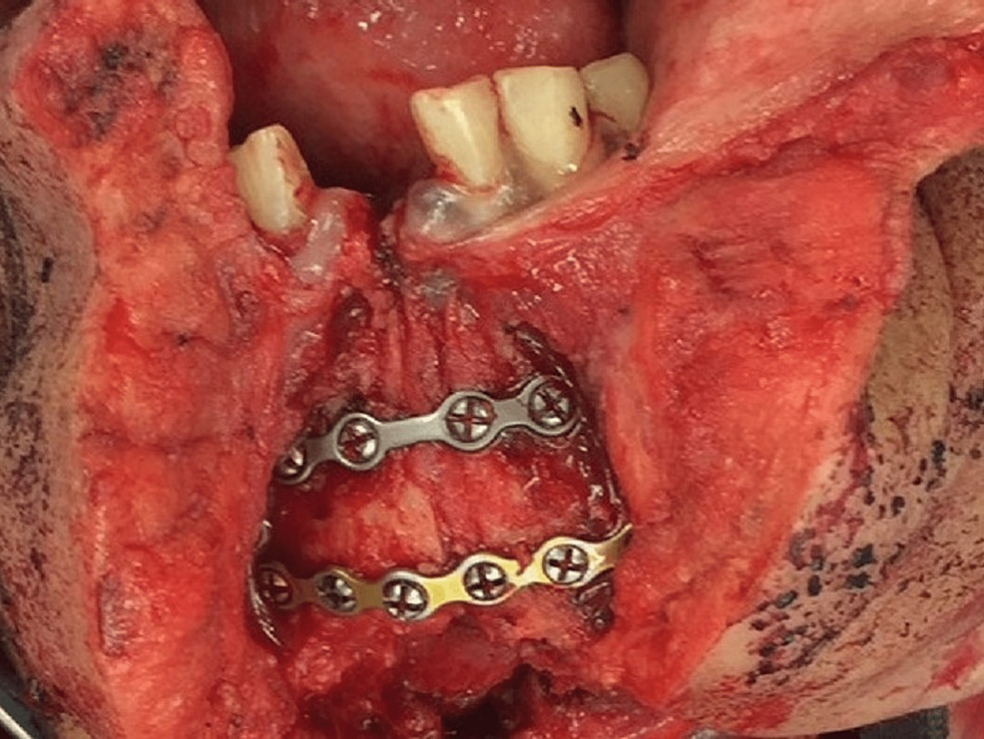
Straight midline mandibulotomy with 2.0-mm miniplate fixation.

Patients were divided into two groups based on mandibular involvement. The first group was composed of patients who underwent oral cancer surgery without mandibular surgery, while the second group was composed of patients who underwent mandibular surgery (mandibulotomy and/or marginal mandibulectomy). All patients in the mandibulotomy group underwent straight midline mandibulotomy and repair with double mini-plates (Figure 1).

**Figure 2:**
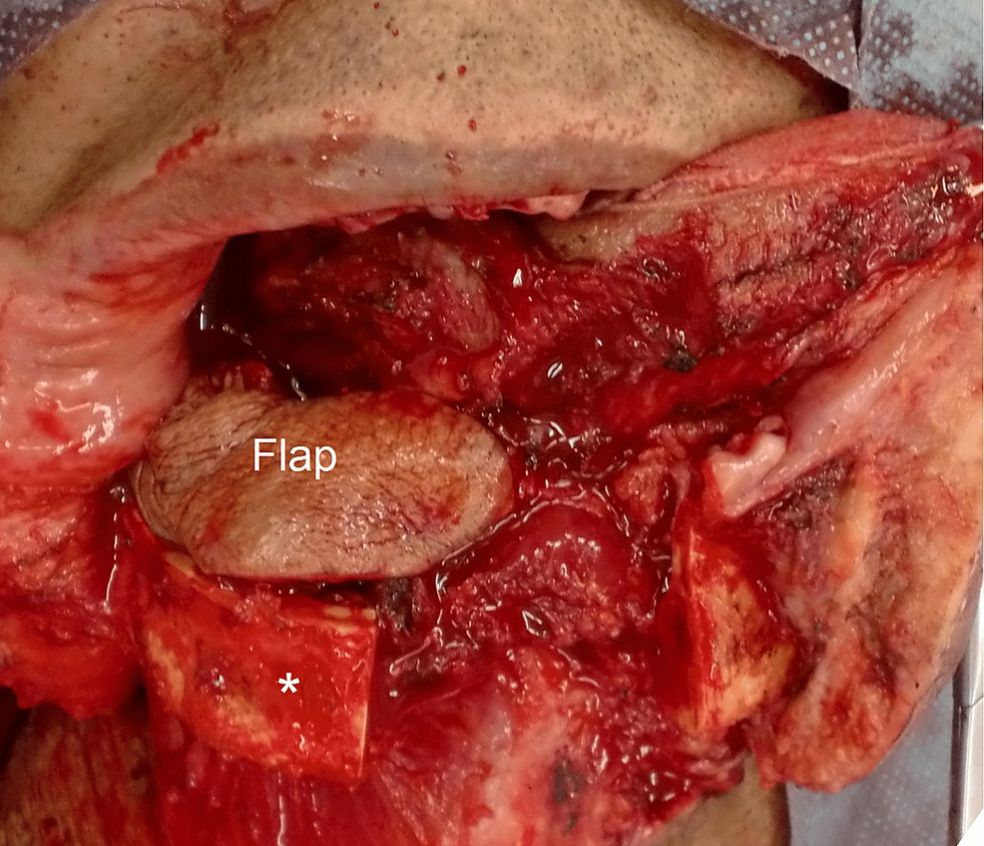
A patient underwent combined midline mandibulotomy and marginal mandibulectomy (*). The infrahyoid myocutaneous flap was placed above the defect.

Marginal mandibulectomies were either performed vertically or horizontally depending on the location of the tumor. Figure 2 shows a patient who underwent combined midline mandibulotomy and marginal mandibulectomy.

Statistical analysis

Comparisons between continuous and categorical data were performed using t-test and Chi-squared test. Cox proportional hazard model was used to assess the association between these factors and the occurrence of osteoradionecrosis. Statistical significance was set at p ≤ 0.05.

Ethical approval

The Chonburi Cancer Hospital ethics committee approved this study and permitted a waiver of informed consent (approval number: 018/65).

## Results

Between 2009 and 2019, from a total of 421 patients with operable oral cancer, 61 patients were included in this study. The reasons for exclusion were surviving less than three years in 154 patients, receiving surgery alone in 112 patients, and loss to follow-up in 39 patients.

The follow-up period was 3-10 years. Since osteoradionecrosis usually occurs late after radiation, only patients who survived for at least three years were included in the analysis. All patients had started dental treatment during surgery and dental problems, such as retained root, extensive periapical lesion, and periodontitis, were completely corrected prior to postoperative radiotherapy.

Postoperative radiotherapy was administered to patients with stage III-IV disease or inadequate margins. Chemotherapy was also used to treat patients with positive margins or adverse features, such as lymphovascular invasion. Concurrent chemotherapy was cisplatin-based and was administered every week at a dose of 40 mg/m2. Chemotherapy was administered for four to six cycles depending on the patient tolerance. The characteristics of the patients included in this study are shown in Table 1.

**Table 1:** Comparison of characteristics of patients with and without mandibular surgery.

Characteristics	Total (n = 61)	Mandibular surgery (n = 32)	No surgery (n = 29)	P-value
Sex (male/female)	34/27	21/11	13/16	0.10
Average age (years)	56.6 (25-77)	56.1 (25–77)	56.8 (40–70)	0.83
Location				
: Tongue	32 (52.5%)	20 (62.5%)	12 (41.4%)	-
: Floor of mouth	8 (13.1%)	7 (21.9%)	1 (3.4%)	
: Buccal, gum, and retromolar trigone	12 (19.7%)	5 (15.6%)	7 (24.1%)	
: Lip and palate	9 (14.8%)	0	9 (31.0%)	
T stage				
: T1–2	38 (62.3%)	16 (50%)	22 (75.9%)	0.04
: T3–4	23 (37.7%)	16 (50%)	7 (24.1%)	
Nodal extension				
: N0	40 (65.6%)	20 (62.5%)	20 (69%)	0.60
: N1–2	21 (34.4%)	12 (37.5%)	9 (31%)	
Stage				
: I–II	26 (42.6%)	8 (25%)	18 (62.1%)	<0.01
: III–IV	35 (57.4%)	24 (75%)	11 (37.9%)	
Radiation dose				
: > 60Gy	30 (49.2%)	16 (50%)	14 (48.3%)	0.99
: 60Gy	27 (44.3%)	14 (43.8%)	13 (44.8%)	
: < 60Gy	4 (6.6%)	2 (6.3%)	2 (6.9%)	
Concurrent chemoradiation	9 (14.8%)	9 (28.1%)	0	<0.01
Smoking	29 (47.5%)	20 (62.5%)	8 (31.0%)	0.01

In our institute, the transoral approach is used for small oral lesions (T1), whereas resection of T4 tumors is usually performed using the mandibulotomy approach. For intermediate oral lesions (T2-3), selection of the approach technique depends on the location of the tumor and the surgeon’s preference and experience. The mandibulotomy approach is usually performed for larger tumors that are difficult to access. This may have led to higher stages of disease in patients who underwent mandibular surgery in this study.

In patients without mandibular surgery, the proportion of patients with lip and buccal cancer was higher. Most patients in this group were elderly women with a history of betel quid chewing. These factors explained the difference in sex and smoking between the groups in Table 1.

Of the 32 patients who underwent mandibular surgery, 11, 15, and six underwent marginal mandibulectomy, mandibulotomy, as well as combined mandibulotomy and marginal mandibulectomy, respectively. Among the 17 patients who underwent marginal mandibulectomy, none experienced postoperative fracture or osteomyelitis.

Among the 21 patients who underwent mandibulotomy, three had mandibulotomy-related complications, one of which had an immediate postoperative wound fistula. The fistula resolved with conservative treatment within three weeks after surgery. The other two patients had plate exposure. These complications were corrected prior to administering radiotherapy. Malunion, non-union, malocclusion, and dental complications were not observed.

The radiotherapy protocol for each patient was conventional two-dimensional radiation with cobalt or a linear accelerator. Among these patients, eleven developed osteoradionecrosis of the mandible. Table 2 shows details of patients with osteoradionecrosis that occurred in this study. The classification by Lyons et al. was used for the evaluation of osteoradionecrosis severity [9]. Only one patient developed severe osteoradionecrosis.

**Table 2:** Details of patients with osteoradionecrosis. *Lyons et al.'s classification of osteoradionecrosis [9]. Stage 1: <2.5 cm length of bone affected; asymptomatic; medical treatment only. Stage 2: >2.5 cm length of bone; asymptomatic, including pathological fracture or involvement of inferior dental nerve or both. Medical treatment only unless there is dental sepsis or obviously loose, necrotic bone. Stage 3: >2.5 cm length of bone; symptomatic, but with no other features despite medical treatment. Consider debridement of loose or necrotic bone, and local pedicled flap. Stage 4: 2.5 cm length of bone; pathological fracture, involvement of inferior dental nerve, or orocutaneous fistula, or a combination. Reconstruction with free flap if patient’s overall condition allows ORN, osteoradionecrosis

No.	Disease	HIV	Surgery group	Onset after radiotherapy	Radiation doses	Chemotherapy	ORN stage*	Location
1. Male 56 years	T3N0 floor of mouth	-	Marginal mandibulectomy	9 years	70Gy	–	1	Symphysis
2. Male 60 years	T2N0 tongue	-	Mandibulotomy	15 months	66Gy	–	1	Body
3. Male 59 years	T3N1 tongue	+	Mandibulotomy	2 years	64Gy	–	1	Body
4. Male 61 years	T3N0 floor of mouth	-	Mandibulotomy with marginal mandibulectomy	2 years	60Gy	–	1	Symphysis
5. Female 60 years	T2N0 tongue	-	No mandibular surgery	2 years	66Gy	–	1	Symphysis
6. Male 67 years	T4N1 tongue	-	Mandibulotomy	6 months	64Gy	–	1	Symphysis
7. Female 54 years	T2N2 tongue	-	Mandibulotomy	2 years	60Gy	Cisplatin weekly	1	Body
8. Female 51 years	T2N1 retromolar trigone	-	Marginal mandibulectomy	1 year	60Gy	Cisplatin weekly	1	Body
9. Male 41 years	T2N0 tongue	+	Mandibulotomy	6 months	64Gy	Cisplatin weekly	1	Symphysis
10. Female 72 years	T4N0 buccal	-	Marginal mandibulectomy	2 years	60Gy	–	2	Body
11. Male 41 years	T1N2 buccal	+	No mandibular surgery	4 years	60Gy	–	4	Body

**Figure 3:**
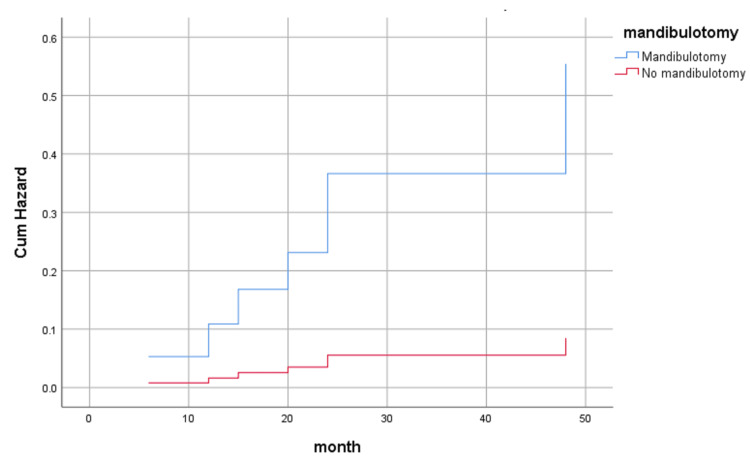
Cumulative hazard plotting of osteoradionecrosis occurrence between patients with mandibulotomy and patients without mandibulotomy.

Table 3 shows the association between osteonecrosis occurrence and risk factors. The significant risk factors for the development of osteoradionecrosis were mandibular surgery, mandibulotomy, and HIV infection. Figure 3 shows the cumulative hazard plotting of osteoradionecrosis of patients with and without mandibulotomy.

**Table 3:** Univariate analysis of risk factors for osteoradionecrosis occurrence. 95%CI, 95% confidence interval; ORN, osteoradionecrosis

Factors	Incidence of ORN (%)	Hazard ratio	95%CI	p-value
Surgical technique				
No mandibular surgery	6.9% (2/29)	reference		
Marginal mandibulectomy and/or mandibulotomy	28.1% (9/32)	4.64	1.002, 21.5	0.05
Mandibulotomy alone	33.3% (5/15)	6.62	1.3, 34.8	0.03
Marginal mandibulectomy alone	27.3% (3/11)	3.56	0.6, 22.0	0.17
Combined marginal mandibulectomy and mandibulotomy	16.7% (1/6)	2.22	0.2, 24.5	0.52
Radiation dose				
≤ 60Gy	16.1% (5/31)	reference		
> 60Gy	20% (6/30)	1.17	0.4, 3.9	0.80
< 60Gy	0% (0/4)	reference		
≥ 60Gy	19.3% (11/57)	22.4	<0.1, >100	0.57
Tumor located near the mandible				
Others	14.6% (6/41)	reference		
Floor of mouth, buccal, gum, and retromolar trigone	25% (5/20)	1.61	0.5, 5.3	0.44
Chemotherapy				
Radiation alone	15.4% (8/52)	reference		
Concurrent chemoradiation	33.3% (3/9)	3.21	0.8, 12.6	0.10
Stage of disease				
Stage I–II	15.4% (4/26)	reference		
Stage III–IV	20% (7/35)	1.54	0.4, 5.4	0.50
Smoking				
No smoking	12.5% (4/32)	reference		
Smoking	24.1% (7/29)	2.32	0.7, 8.0	0.18
Human immunodeficiency virus infection				
No infection	14.0% (8/57)	reference		
Infection	75% (3/4)	8.53	2.2, 33.3	<0.01
Diabetes mellitus				
No diabetes mellitus	18.3% (11/60)	reference		
Diabetes mellitus	0% (0/1)	0.05	<0.1, >100	0.79
Age				
Age < 60 years	20.6% (7/34)	reference		
Age ≥ 60 years	14.8% (4/27)	0.63	0.2, 2.2	0.48

In subgroup analysis, marginal mandibulectomy was nonsignificantly associated with the development of osteoradionecrosis. Moreover, other factors, such as tumor concurrent chemoradiation, radiation dose ≥ 60Gy, and smoking, showed hazard ratios of two or more but none of these factors were statistically significant.

Table 4 shows multivariate analysis of the association between osteonecrosis occurrence and risk factors. Mandibular surgery, mandibulotomy, and HIV infection remain significantly associated with the development of osteoradionecrosis.

**Table 4:** Multivariate analysis of the development of osteoradionecrosis.

Factors	Adjusted hazard ratio	95%CI	p-value
Mandibular surgery (adjusted with HIV infection)	8.30	1.5, 44.7	0.01
HIV infection (adjusted with mandibular surgery)	18.6	3.9, 88.4	<0.01
Mandibulotomy (adjusted with HIV infection)	9.13	1.5, 55.4	0.02

## Discussion

Osteoradionecrosis is one of the most serious oral complications following head and neck cancer treatment. It is characterized by bone tissue necrosis and failure to heal for at least three months [10]. Although the precise pathogenic mechanisms are not fully understood, osteoradionecrosis of the mandible is caused by radiation-induced cellular injury, ultimately resulting in a chronic, nonhealing wound [6,10].

Several factors, such as poor dental health, post-radiation dental extractions, radiation doses, smoking and alcohol use, tumor locations (oropharynx vs oral cavity), proximity of the tumor to the bone, and nutritional status, have been implicated as risk factors for the development of osteoradionecrosis [6,10-12].

While previous studies suggested that mandibular surgery during oral cancer operation might increase the risk of osteoradionecrosis, to date, few studies have demonstrated a statistically significant association between mandibular surgery and osteoradionecrosis occurrence [5,12,13]. This study aimed to assess the occurrence of osteoradionecrosis and other complications related to mandibular surgery in patients with oral cancer. The relationship between mandibular surgery and the development of osteoradionecrosis was analyzed.

The mandibulotomy approach is associated with increased complications, such as wound infection, plate exposure, and osteoradionecrosis [8]. Previous studies have reported complication rates between 11% and 46.5% [8,14,15]. Complications related to mandibulotomy that occurred in this study were plate exposure and wound fistula. These complications were minor and within acceptable levels. None of the patients who underwent marginal mandibulectomy experienced postoperative fracture or osteomyelitis.

The overall incidence rate of osteoradionecrosis in this study was 18.0%. This was similar to the rates from other studies using the same two-dimensional radiotherapy technique, which ranged from 5% to 20% [16]. Recent advances in radiotherapy such as three-dimensional conformal radiotherapy or intensity-modulated radiotherapy can further reduce the risk of osteoradionecrosis [17].

The extent to which mandibular surgery influences the occurrence of osteoradionecrosis remains unclear. The analysis of risk factors for osteoradionecrosis may have various results, depending on population selection, types and stages of tumors, radiotherapy protocol, and duration of study. The existence of confounding variables in previous studies makes it difficult to establish a clear causal link between mandibular surgery and osteoradionecrosis occurrence [4,7-12].

In this study, confounding factors from the site of tumor, oral hygiene, and nutrition were minimized because only patients with oral cancer were included. In addition, the hospital treatment protocol required all patients to correct their oral hygiene and nutrition prior to surgery and radiotherapy. Unlike previous studies that might include various protocols of radiotherapy and conditions of patients, the treatment in this study was surgery and postoperative radiotherapy with curative intent for every patient [10,12,18].

The univariate and multivariate analyses revealed that mandibular surgery and mandibulotomy were significantly related to the occurrence of osteoradionecrosis. However, the study is underpowered to clarify the role of marginal mandibulectomy as an independent risk factor for developing osteoradionecrosis.

Our study also found that patients with HIV infection showed a significant increase in the occurrence of osteoradionecrosis. HIV-infected patients are at a higher risk of developing osteonecrosis disease due to vascular compromise. This may be related to the HIV disease itself or its therapy [19]. To our knowledge, this finding is the first to report the association in patients receiving radiotherapy. However, because only four patients with HIV were included in this study, this finding requires further confirmation.

The primary limitation of this study is the inherent selection bias and confounders associated with the retrospective nature of this study. Analysis of risk factors for osteoradionecrosis may have limited the validity. Osteoradionecrosis is a late complication and many patients died or were lost to follow-up prior to the occurrence of osteoradionecrosis. The incidence rate of osteoradionecrosis varies depending on population selection. Although patient factors were minimized, as only patients with oral cancer were included, the relatively small number of cases included in this study limited the statistical significance of several parameters.

## Conclusions

The findings of this study suggest that mandibular surgery, especially in patients undergoing mandibulotomy, was significantly associated with the occurrence of osteoradionecrosis. Preventive therapies, such as the maintenance of meticulous oral hygiene and frequent visits to the dentist, should be the key focus for these patients. However, further studies including larger population sizes are required to verify these findings.
